# The Interplay of RecA-related Proteins and the MND1–HOP2 Complex during Meiosis in Arabidopsis thaliana

**DOI:** 10.1371/journal.pgen.0030176

**Published:** 2007-10-12

**Authors:** Julien Vignard, Tanja Siwiec, Liudmila Chelysheva, Nathalie Vrielynck, Florine Gonord, Susan J Armstrong, Peter Schlögelhofer, Raphael Mercier

**Affiliations:** 1 Station de Génétique et d'Amélioration des Plantes, INRA, Versailles, France; 2 Department of Chromosome Biology, Max F. Perutz Laboratories, University of Vienna, Vienna, Austria; 3 School of Bioscience, the University of Birmingham, Birmingham, United Kingdom; Stowers Institute for Medical Research, United States of America

## Abstract

During meiosis, homologous chromosomes recognize each other, align, and exchange genetic information. This process requires the action of RecA-related proteins Rad51 and Dmc1 to catalyze DNA strand exchanges. The Mnd1–Hop2 complex has been shown to assist in Dmc1-dependent processes. Furthermore, higher eukaryotes possess additional RecA-related proteins, like XRCC3, which are involved in meiotic recombination. However, little is known about the functional interplay between these proteins during meiosis. We investigated the functional relationship between AtMND1, AtDMC1, AtRAD51, and AtXRCC3 during meiosis in Arabidopsis thaliana. We demonstrate the localization of AtMND1 to meiotic chromosomes, even in the absence of recombination, and show that AtMND1 loading depends exclusively on AHP2, the *Arabidopsis* Hop2 homolog. We provide evidence of genetic interaction between *AtMND1*, *AtDMC1*, *AtRAD51*, and *AtXRCC3.* In vitro assays suggest that this functional link is due to direct interaction of the AtMND1–AHP2 complex with AtRAD51 and AtDMC1. We show that AtDMC1 foci accumulate in the *Atmnd1* mutant, but are reduced in number in *Atrad51* and *Atxrcc3* mutants. This study provides the first insights into the functional differences of AtRAD51 and AtXRCC3 during meiosis, demonstrating that AtXRCC3 is dispensable for AtDMC1 focus formation in an *Atmnd1* mutant background, whereas AtRAD51 is not. These results clarify the functional interactions between key players in the strand exchange processes during meiotic recombination. Furthermore, they highlight a direct interaction between MND1 and RAD51 and show a functional divergence between RAD51 and XRCC3.

## Author Summary

During meiosis, two rounds of chromosome segregation follow a single round of chromosome duplication, leading to the production of haploid gametes. It is during this specialised cell division that genetic traits are recombined, achieved by mutual exchange of DNA sequences of homologous chromosomes. Recombination events must be well controlled to ensure that: (i) they occur between homologous chromosomes rather than sister chromatids and (ii) they occur between true homologous sequences and not duplicated sequences present elsewhere in the genome. At one of the early steps of homologous recombination, a single-strand DNA molecule recognizes and invades the targeted homologous sequence. Many proteins, which are conserved throughout the kingdoms, are involved in this crucial step of DNA homology search. In this study, we analyze the role of a series of such proteins and their complex functional relationships in the model plant A. thaliana, shedding new light on this highly regulated step of meiotic recombination.

## Introduction

In sexual organisms, ploidy must be reduced before fertilization to maintain the same number of chromosomes over different generations. The parental genetic material is reduced at meiosis—a specialized cell division, in which a single round of DNA replication is followed by two rounds of chromosome segregation. The orderly disjunction of homologous chromosomes at the first meiotic division requires their prior pairing. In most species, homologous recombination ensures that the two homologous chromosomes interact physically. Strand exchange between homologous sequences, a crucial step in recombination, involves RecA-related proteins [1]. The choice of DNA substrate must be well controlled to ensure that recombination occurs between homologous chromosomes rather than between the sister chromatids, and between allelic rather than ectopic sequences.

**Figure 1:**
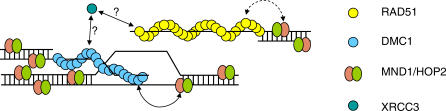
Putative Model of the Strand Invasion Step of Meiotic Recombination The diagram represents a model of the strand invasion step during meiotic recombination. Several key players are represented with their putative functional interactions (arrows). DMC1 may assemble on one of the single-stranded DNA strands after processing of a DSB, whereas RAD51 may assemble on the other accessible single strand. In this model, the DMC1 nucleoprotein filament invades the homologous chromosome, being assisted by the MND1/HOP2 complex. RAD51 may also be stimulated by the HOP2/MND1 complex (dashed arrow) in the strand exchange processes. XRCC3 seems to be involved at this step but its role and interactions remain unclear [3,13,69].

The molecular basis of meiotic recombination has been well studied in budding yeast. It is initiated by programmed double-strand breaks (DSBs), catalyzed by the Spo11 protein [2]. DNA processing at the sites of DSBs generates single-stranded tails, which may be loaded with DNA strand–exchange proteins to form nucleoprotein filaments. These DNA–protein filaments are thought to be involved in active homology searches and strand exchanges—a prerequisite for the identification of homologous chromosomes and DSB repair [3]. Furthermore, meiotic DSBs are required for formation of the synaptonemal complex (SC) and the generation of crossovers. Two recombinases are known to form presynaptic nucleoprotein filaments with single-stranded DNA during meiosis: the RecA homologs Rad51 and Dmc1 [4,5]. Rad51 is involved in both mitotic and meiotic recombination, whereas Dmc1 is involved only in meiosis and seems to have a specific role in recombination between homologs [6]. The two RecA homologs play unique, different roles during meiotic DSB repair. Nevertheless, they also cooperate to achieve efficient meiotic recombination, presumably by asymmetric assembly at either end of the DSB [3] (Figure 1).

Other RecA-related proteins are found in eukaryotes. For instance, Rad55 and Rad57 seem to be specific to yeast, whereas vertebrates have five Rad51 paralogs: RAD51B, RAD51C, RAD51D, XRCC2, and XRCC3 [7]. Rad55 and Rad57 interact with Rad51 and are involved in Rad51-dependent meiotic recombination in yeast [8,9]. In higher eukaryotes, two stable complexes involving RAD51 paralogs have been purified biochemically: the first includes RAD51B, RAD51C, RAD51D, and XRCC2, and the second includes RAD51C and XRCC3 [10]. Mammalian RAD51 paralogs work together with RAD51 during somatic DNA repair, but their role in meiotic recombination remains unclear, due to the lethality of knockout mutants. Recently, a hypomorphic mutation of the RAD51C gene has been generated in mice affecting both male and female meiosis [11].

The heterodimeric Mnd1-Hop2 complex has also been shown to be absolutely required for the meiotic recombination processes. In *mnd1* and *hop2* budding yeast mutants, meiosis arrests before the first division, DSBs are not repaired, and almost complete synapsis occurs between nonhomologous chromosomes [12–14]. Mouse *Hop2* knockout mutants are also deficient in meiotic DSB repair, but, in contrast to budding yeast mutants, display limited synapsis [15]. In Saccharomyces cerevisiae, the Mnd1-Hop2 complex assists in a Dmc1-dependant pathway during homologous recombination, as it can promote Dmc1 strand assimilation activity in vitro [16]. The absence of Mnd1 or Hop2 leads to the accumulation of both Dmc1 and Rad51, although various studies have suggested that the Mnd1–Hop2 complex is active only in a Dmc1-dependent pathway in yeast [12,14,17]. First, the phenotype of either the *mnd1* or *hop2* mutant is very similar to that of *dmc1* and different from that of *rad51* [13,16]. Second, the meiotic defects of *mnd1*, *hop2*, or *dmc1* mutants can be bypassed by overexpressing *RAD51* [18]. Finally, no effect of Mnd1-Hop2 on Rad51 activity has ever been reported in yeast. The idea that DMC1, MND1, and HOP2 form a functional unit is supported by the fact that nematodes, fruit flies, and Neurospora crassa lack not only a gene encoding a *DMC1* homolog but also genes encoding *HOP2* or *MND1* homologs*.* However, in mammals, Mnd1-Hop2 complexes can interact with Dmc1 but also with Rad51, stimulating the activities of both proteins in vitro [19–22] (Figure 1).

In recent years, efforts have focused on clarifying meiotic mechanisms in A. thaliana. The apparent absence of strict meiotic checkpoints and the viability of several *Arabidopsis* mutants, as opposed to the lethality of the corresponding mutations in mammals, have made this plant an ideal model organism to apply powerful genetic and cytological approaches. Two Spo11 homologs *AtSPO11–1* and *AtSPO11–2* are essential for initiation of meiotic recombination [23,24]. Furthermore, homologs of Rad51 and Dmc1 have been identified, and characterization of the corresponding mutants has revealed important differences in their role during meiosis. *Atrad51* mutants fail to repair meiotic DSBs, as shown by extensive *AtSPO11–1*-dependent chromosome fragmentation during meiosis [25]. In contrast, the chromosomes in *Atdmc1* mutants do not fragment but segregate as univalents during meiosis I [26]. The formation of nonfragmented univalents in *Atdmc1* is dependent on *AtRAD51* and it is thought that the DSBs formed in *Atdmc1* mutants are repaired via the sister chromatid [27]. Disruption of *AHP2* (the *Arabidopsis Hop2* homolog) or *AtMND1* leads to meiotic defects similar to those observed in *Atrad51* mutants but not to those in *Atdmc1* mutants [28,29]. AtMND1 function seems to be required after recombinase assembly because, as in yeast, AtRAD51 foci are seen in *Atmnd1* mutants [28]. In addition to *AtRAD51* and *AtDMC1*, the five *RAD51* paralogs identified in vertebrates are also present in the *Arabidopsis* genome [30]. *AtRAD51B*, *AtRAD51C*, *AtXRCC2*, and *AtXRCC3* are required for DNA repair, but only the products of *AtRAD51C* and *AtXRCC3* are involved in meiosis [31,32]. Phenotypic analyses of *Atrad51c* and *Atxrcc3* mutants have shown that, as in *Atrad51*, *Atmnd1*, and *ahp2* mutants, chromosome fragmentation occurs without prior chromosome synapsis. All the proteins cited above (together with some others, e.g., *AtBRCA2* [27]) are required for correct DSB repair, chromosome pairing, and synapsis. However, little is known about their functional relationship and their genetic and physical interactions in *Arabidopsis*: AtBRCA2 interacts with either AtDMC1 or AtRAD51 [33], and the repair of meiotic DSBs in *Atdmc1* mutants is dependent on AtBRCA2 and AtRAD51 [27]. Two-hybrid assays have shown that AtMND1 interacts with AHP2 and that AtXRCC3 interacts with AtRAD51 and AtRAD51C [28,34].

In this study, we investigated in detail the meiotic function of AtMND1 and its interactions (genetic and physical) with the RecA-related proteins AtRAD51, AtDMC1, and AtXRCC3. As in yeast, AtMND1 was localized along the entire length of the chromosome and required AHP2 for loading onto chromosomes. However, its loading did not depend on the initiation of recombination, DSB processing, or cohesion. Despite the lack of direct evidence for the colocalization of AtMND1 and AtDMC1, we demonstrated that AtMND1 is required for normal AtDMC1 distribution. We also investigated the interdependence of AtMND1, AtDMC1, AtRAD51, and AtXRCC3 in meiotic DSB repair and AtDMC1 loading. We showed for the first time a genetic interaction between MND1 and RAD51. We also provided data for direct interactions of AtMND1 and AHP2 with both AtDMC1 and AtRAD51 in vitro. We showed that AtRAD51 is required for efficient AtDMC1 foci formation in an *Atmnd1* mutant background, whereas AtXRCC3 is not. This study dissected the differential function of individual RecA-related homologs during meiotic DSB repair, and notably points out a functional divergence between RAD51 and XRCC3.

## Results

### AtMND1 Is Localized to Chromatin during Meiosis

**Figure 2:**
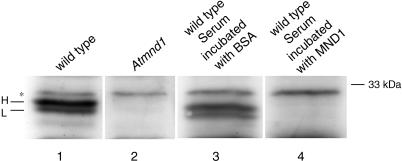
Western Blot with the AtMND1 Antibody The AtMND1 antibody yields two specific bands when applied to blotted protein extracts from wild-type buds, designated “H” and “L,” for AtMND1 species with “higher” (29.8 kDa) and “lower” (29 kDa) molecular mass (Lane 1). These bands were not detected in the *Atmnd1* mutant (Lane 2). Furthermore, no such bands were detected when the serum was depleted of the specific AtMND1 antibody by preincubation of the serum with recombinant AtMND1 protein (Lane 4). The AtMND1 antibody was not depleted by incubation of the serum with BSA (Lane 3). The asterisk designates a nonspecific band. The preimmune serum did not recognize these two protein species (unpublished data).

**Figure 3:**
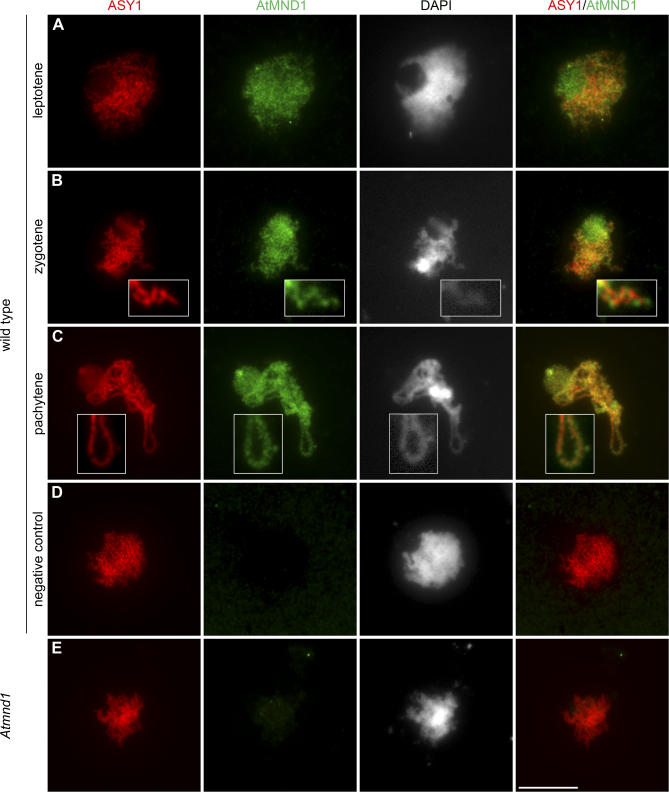
Immunolocalization of the AtMND1 Protein Male meiocytes of the wild type (A–D) and the *Atmnd1* mutant (E). Chromosomes are stained with the ASY1 antibody (red), the AtMND1 antibody (green), and DAPI (gray). The AtMND1 antibody strongly labels the chromosomes and nucleolus in the wild type (B), whereas no signal is detected in *Atmnd1* mutants (E). The signal disappears also in wild type when the serum was preincubated with the recombinant AtMND1 protein (D) but was not affected when the serum was preincubated with BSA (unpublished data). Scale bar, 10 μm.

We investigated the distribution of the AtMND1 protein during meiosis by immunolocalization in wild-type meiotic cells, using a polyclonal antibody against AtMND1. ASY1, an axial element protein, was used as a marker of meiotic progression [35]. The specificity of the MND1 antibody was demonstrated in both western blot and cytology experiments by comparing wild-type and *Atmnd1* mutant plants. The antibody did not result in a signal from mutant plant material following either experimental approach (Figures 2 and 3). Moreover, the signals observed in wild-type plant material disappeared in both western blot and cytology experiments when the serum was preincubated with the recombinant AtMND1 protein, confirming the specificity of the serum (Figures 2 and 3D). Meiotic chromosomes from wild-type plants were strongly labeled with anti-AtMND1 antibody (Figure 3A–3C). AtMND1 was first detected at early leptotene, when ASY1 filaments had not yet completely formed along the chromosomal axes (Figure 3A). AtMND1 was also detected in the nucleolus. Several data suggest that the nucleolus may function as a reservoir of proteins, including proteins involved in DNA repair and meiosis [36,37]. A pool of AtMND1 protein may thus be stocked in the nucleolus. As meiosis progressed and the ASY1 signal extended all along the axial element, AtMND1 was detected along the entire length of the chromosome, in both unsynapsed and synapsed chromosome regions (Figure 3B and 3C). The MND1 labeling was thicker than the one for ASY1, for which the signal was detected exclusively along the axis of the chromosomes. Thus, in clear contrast to ASY1, AtMND1 was localized to both chromosome axes and loop regions (Figure 3B and 3C). Regions of lower and higher intensity of AtMND1 signal were detected, the higher-intensity regions resembling foci.

### The Distribution of AtMND1 Depends on AHP2, but Not on the Initiation of Recombination or Establishment of Cohesion

**Figure 4:**
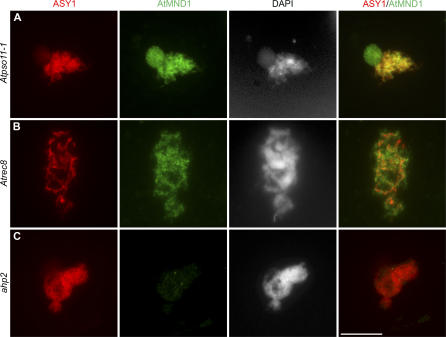
The Distribution of AtMND1 Depends on AHP2, but Not on AtSPO11–1 or AtREC8 Male meiocytes of *atspo11–1* (A), *Atrec8* (B), and *ahp2* (C) mutants. Chromosomes are stained with the ASY1 antibody (red), the AtMND1 antibody (green), and DAPI (gray). Magnified images of individual chromosome axes are shown within the white rectangles. No AtMND1 signal is detected in *ahp2* meiocytes, whereas the distribution of AtMND1 appears to be normal in *Atspo11–1* and *Atrec8*. Scale bar, 10 μm.

We analyzed the distribution of AtMND1 in several mutants. We investigated AtMND1 loading in mutants with disrupted meiotic recombination initiation (*Atspo11–1*), DSB processing (*Atmre11*), strand invasion, and homology search (*Atrad51*, *Atxrcc3*, and *Atdmc1*), and SC formation (*asy1*) [23,25,26,31,38,39]. The distribution of AtMND1 was not affected in these six mutants (Figures 4A and S1), showing that the localization of AtMND1 is independent of the above-mentioned processes. We also investigated the distribution of AtMND1 in *Atscc3* and *Atrec8* mutants, to determine whether cohesion was required for AtMND1 loading on chromosomes [40,41]. We observed no aberration of AtMND1 distribution in these mutants (Figures 4B and S1F). These results indicate that AtMND1 is present on meiotic chromosomes during meiosis, even in the absence of recombination, axis formation, or cohesion. This finding was confirmed by the normal AtMND1 distribution in *swi1* mutants (Figure S1G), in which meiotic recombination, the establishment of cohesion, and the formation of axial elements are defective [42,43].

We investigated whether AtMND1 distribution depended on *AHP2*, the *Arabidopsis* homolog of Hop2 [29]. AtMND1 was not detected on the meiotic chromosomes of *ahp2* mutants (Figure 4C), demonstrating the crucial role of AHP2 in controlling the distribution of AtMND1.

### AtMND1 and AtDMC1 Are Not Colocalized

**Figure 5:**
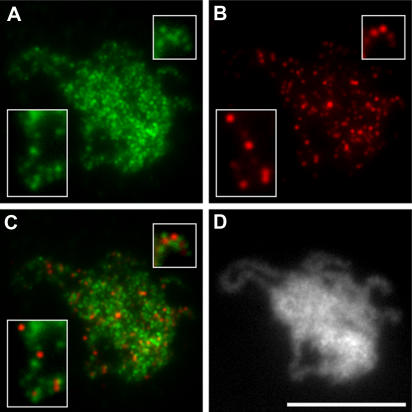
AtMND1 and AtDMC1 Are Not Colocalized Wild-type male meiocyte stained with the AtMND1 antibody (A), the AtDMC1 antibody (B), and DAPI (D). (C) was generated by merging A and B. AtDMC1 foci are not extensively co-localized with AtMND1 on meiotic chromosomes. Magnified images of individual chromosome axes are shown within the white rectangles. Scale bar, 10 μm.

We investigated the possibility of AtMND1/AtDMC1 colocalization in *Arabidopsis*, by coimmunolocalization studies on meiocyte spreads. AtDMC1 foci overlapped with the diffuse signals for AtMND1, but the regions strongly labeled for AtMND1 displayed no colocalization with the AtDMC1 foci (Figure 5).

### Relationships between *AtMND1*, *AtDMC1*, *AtRAD51*, and *AtXRCC3*

In almost all organisms, including A. thaliana, the Rad51 and Dmc1 recombinases are both required for meiotic recombination to be performed correctly [3,26,44]. In addition, *Arabidopsis* has five RAD51 paralogs in its genome, all of which have homologs in mammalian genomes. Only two of these paralogs, *AtRAD51C* and *AtXRCC3*, have been shown to encode proteins with meiotic function [31,32]. We investigated the links between AtMND1 and RecA-related proteins in *Arabidopsis*, by studying the epistatic relationships between *Atmnd1*, *Atrad51*, *Atdmc1*, and *Atxrcc3,* and by analyzing the phenotypes of the corresponding double and triple mutants.

**Figure 6:**
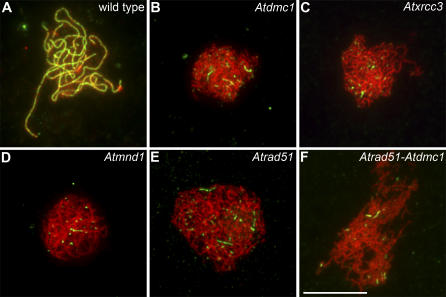
Synapsis Is Impaired in *Atdmc1*, *Atxrcc3*, *Atmnd1*, *Atrad51*, and *Atrad51-Atdmc1* Mutants Male meiocytes stained with the ASY1 antibody (red) and the AtZYP1 antibody (green). During wild-type pachytene stages AtZYP1 extends along the entire length of the axes (A). In *Atdmc1* (B), *Atxrcc3* (C), *Atmnd1* (D), *Atrad51* (E), and *Atrad51-Atdmc1* (F) mutants, AtZYP1 staining is restricted to a few foci and a few short stretches. Scale bar, 10 μm.

In the wild type, homologous chromosomes synapse along their entire length at pachytene (Figure S2A). At this stage, a fully extended SC can be visualized by immunolocalization of the AtZYP1 protein, which forms the transverse filament of the SC [45], coupled with immunolocalization of ASY1, to label the chromosome axes [35] (Figure 6A). The first visible defect in *Atrad51* and *Atmnd1* mutants, which display similar meiotic phenotypes [25,28], is the absence of a normal pachytene stage. This stage is replaced by a failed pachytene stage with unsynapsed chromosomes (Figure S2B and S2C). In *Atdmc1* mutants, the chromosomes also fail to synapse (Figure S2D). In the *Atxrcc3* mutant, synapsis has been reported [31]; however, we observed exclusively failed pachytene stages (n = 125) and never synapsed chromosomes in this mutant (Figure S2E). We clarified the issue of synapsis by immunolocalizing AtZYP1 in *Atdmc1*, *Atxrcc3*, *Atmnd1,* and *Atrad51* mutants. The *Atdmc1* mutant has been shown to be defective in extensive AtZYP1 localization [45] (Figure 6B). AtZYP1 is observed only as numerous foci on chromosome axes, a minority of which elongate to form short filaments. A similar pattern of staining was observed in this study for AtZYP1 in *Atxrcc3*, *Atmnd1*, and *Atrad51* mutants (Figure 6C–6E), demonstrating that synapsis is impaired in all these mutants. A similar type of AtZYP1 loading was also observed when both recombinases, AtRAD51 and AtDMC1, were disrupted (Figure 6F).

**Figure 7:**
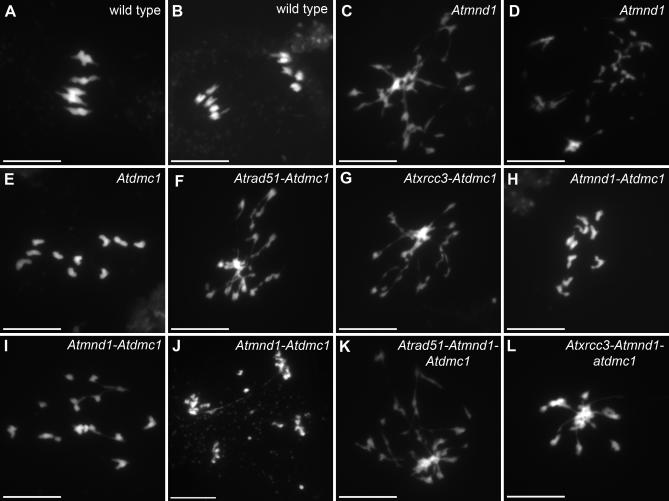
Epistatic Relationships between *AtMND1*, *AtRAD51*, *AtXRCC3*, and *AtDMC1* In wild type, five bivalents are aligned at metaphase I (A), and the homologous chromosomes segregate at anaphase I (B). In *Atmnd1* (C), as in *AtRad51* and *Atxrcc3* (see Figure S2), an entangled mass of chromosomes is observed at metaphase I, with chromatid connections between multiple chromosomes. Segregation at anaphase I leads to chromosome fragmentation (D). In *Atdmc1*, ten univalents are visible at metaphase I (E). Typical *Atrad51* or *Atxrcc3* single mutant metaphase I defects are seen in *Atrad51-Atdmc1* (F) and *Atxrcc3-Atdmc1* (G) double mutants, respectively. Metaphase I defects in *Atmnd1-Atdmc1* mutants are intermediate between those of the single mutants: some meiocytes have ten univalents (H), whereas the others display chromatin links and fragmentation (I). In most *Atmnd1-Atdmc1* anaphase II chromatid connections and fragmentation following chromatid separation were observed (J). The mutation of either *AtRAD51* (K) or *AtXRCC3* (L) in the *Atmnd1-Atdmc1* mutant results in a typical *Atrad51* meiosis defect. Scale bar, 10 μm.

During metaphase I in wild type, five bivalents align, allowing homologous chromosomes to segregate properly at anaphase I (Figure 7A and 7B). *Atmnd1*, *Atrad51*, and *Atxrcc3* mutants do not undergo classical metaphase I with the alignment of bivalents; instead, they display entangled chromosomes interconnected by chromatin links [25,28,31] (Figures 7C and S3). Then the chromosomes start to separate at anaphase I, leading to chromosome fragmentation (Figures 7D and S3). We generated the associated double mutants and showed identical phenotypes compared to the single mutants (unpublished data). Taking advantage of the difference that the *Atdmc1* mutant displays compared to *Atmnd1*, *Atrad51*, and *Atxrcc3* at prophase end and afterward, we investigated the meiotic behavior of *Atmnd1*, *Atrad51*, and *Atxrcc3* mutants in the *Atdmc1* background to decipher the relationship between these factors. The chromosomes of the *Atdmc1* mutant are not aligned as bivalents at metaphase I but instead, they remain as intact univalents [26] (Figure 7E), resulting in random segregation at anaphase I [26]. The metaphase–anaphase I stages of the double *Atrad51-Atdmc1*, and *Atxrcc3-Atdmc1* mutants resembled those of the *Atrad51* and *Atxrcc3* single mutants (Figure 7F and 7G), with the same pattern of chromosome entanglement followed by fragmentation. Thus, *AtRAD51* and *AtXRCC3* are epistatic to *AtDMC1*. In contrast, the *Atmnd1-Atdmc1* double mutant had intermediate meiotic defects between those of the two single mutants. Its phenotype varied from meiocytes with ten apparently intact univalents (42%, n = 220) during metaphase-anaphase I (Figure 7H) to meiocytes with fewer chromatid links and milder chromosome fragmentation than the *Atmnd1* mutant (Figure 7I). However, fragmentation defects and the abundance of chromatid links were obvious at the second division, after sister chromatid separation (Figure 7J). Indeed, a larger proportion of meiocytes (98%, n = 57) presented fragmented chromosomes during the second division than during the first. Seed counts with the *Atmnd1-Atdmc1* double mutant showed that seed formation rates were similar to those for the *Atmnd1* single mutant, and significantly lower than those for the *Atdmc1* single mutant (unpublished data).

We also studied meiotic chromosomes of the *Atrad51-Atdmc1-Atmnd1* and *Atdmc1-Atmnd1-Atxrcc3* triple mutants*.* The metaphase–anaphase I phenotype of these mutants is similar to that of the *Atmnd1*, *Atrad51*, and *Atxrcc3* single mutants (Figure 7K and 7L). Thus, the depletion of *AtRAD51* or *AtXRCC3* in the *Atmnd1-Atdmc1* background results in a reversion to a defect resembling the *Atmnd1*, *Atrad51*, or *Atxrcc3* single-mutant defect. In conclusion, the disruption of *AtMND1*, *AtRAD51*, or *AtXRCC3* leads to the same meiotic phenotype. The formation of intact univalents during prophase I in *Atdmc1* mutants is only mildly affected by the *Atmnd1* mutation, but absolutely requires AtRAD51 or AtXRCC3. We deduce that *AtRAD51* and *AtXRCC3* are epistatic to *AtDMC1*, and to *AtMND1*, in an *Atdmc1* mutant background.

### AtDMC1 Accumulates on Meiotic Chromosomes in *Atmnd1* Mutants

**Figure 8:**
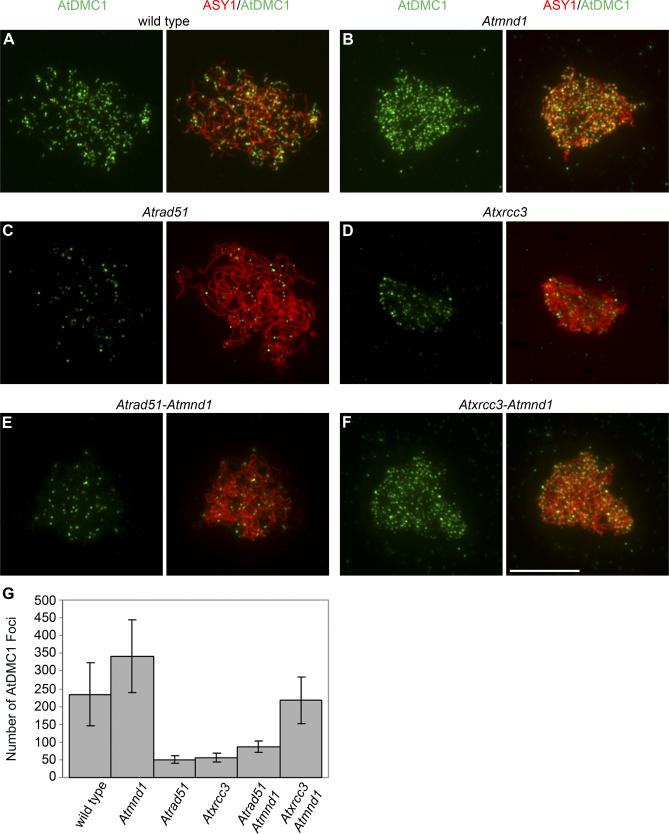
Efficient AtDMC1 Loading Requires the Presence of AtRAD51, But Not of AtXRCC3, in the *Atmnd1* Mutant Background Distribution of AtDMC1 foci in male meiocytes of the wild type (A), *Atmnd1* (B), *Atrad51* (C), *Atxrcc3* (D), *Atrad51-Atmnd1* (E), and *Atxrcc3-Atmnd1* (F) mutants. Chromosomes have been stained with the ASY1 antibody (red) and the AtDMC1 antibody (green). The AtDMC1-only (left) and the AtDMC1–ASY1 merge (right) images are shown. AtDMC1 foci are localized on the chromosome axes, as revealed by the ASY1 signal. The mean number of AtDMC1 foci for the wild type and all mutants is presented in the histogram (G). Scale bar, 10 μm.

As the Mnd1–Hop2 complex appears to be active in a Dmc1-dependent pathway (see Introduction), we investigated the distribution of AtDMC1 foci in *Atmnd1* meiocytes. An antibody directed against a synthetic peptide specific for the AtDMC1 protein [46] was used in immunolocalization experiments, together with an antibody against ASY1 [35]. We analyzed the number of AtDMC1 foci on wild-type chromosomes during meiotic progression and obtained results similar to those reported by [46], with the number of AtDMC1 foci maximal during zygotene (Figure 8A). The mean number of AtDMC1 foci in the wild type was 234 ± 89 (n = 28). We counted AtDMC1 foci in *Atmnd1* cells during the failed pachytene stage replacing the wild-type zygotene-pachytene stages (Figure 8B). We observed a mean of 342 ± 103 (n = 22) foci, a number significantly higher than that for the wild type (*p* < 10^−4^; custom hypothesis tested with a contrast using the GLM procedure of SAS 8.1; SAS Institute). Thus, AtDMC1 foci accumulate in the *Atmnd1* mutant, indicating a failure to progress in the meiotic strand invasion process.

### The Accumulation of AtDMC1 Foci in *Atmnd1* Depends on *AtRAD51* but Not on *AtXRCC3*

We analyzed the number of AtDMC1 foci in *Atrad51* and *Atxrcc3* mutants. The distribution of AtDMC1 was considerably altered in these mutants (Figure 8C and 8D), with a mean of 50±11 (n = 21) foci in the *Atrad51* mutant and 56±13 (n = 17) in the *Atxrcc3* mutant (no significant difference between these two results; *p* = 0.81). We conclude that the AtRAD51 and AtXRCC3 proteins are required for efficient AtDMC1 foci formation.

We therefore analyzed the number of AtDMC1 foci in *Atrad51-Atmnd1* and *Atmnd1-Atxrcc3* double mutants (Figure 8G). The *Atrad51-Atmnd1* double mutant had far fewer AtDMC1 foci than the *Atmnd1* single mutant (Figure 8E), with a mean of 87±16 foci (n = 17). This number of foci is significantly different from that for the *Atmnd1* single mutant (*p* < 10^−4^) or the wild type (*p* < 10^−4^). The accumulation of AtDMC1 foci in *Atmnd1* mutants therefore depends on *AtRAD51*. In contrast, the mean number of AtDMC1 foci in the *Atmnd1-Atxrcc3* double mutant (Figure 8F, 217±76; n = 35) is only slightly lower than that for the *Atmnd1* single mutant (*p* < 10^−4^). Thus, AtDMC1 foci formation is not dependent on AtXRCC3 in an *Atmnd1* mutant background and we conclude that AtRAD51 and AtXRCC3 play different roles during meiosis.

### AtMND1 and AHP2 Interact with AtRAD51 and AtDMC1

**Figure 9:**
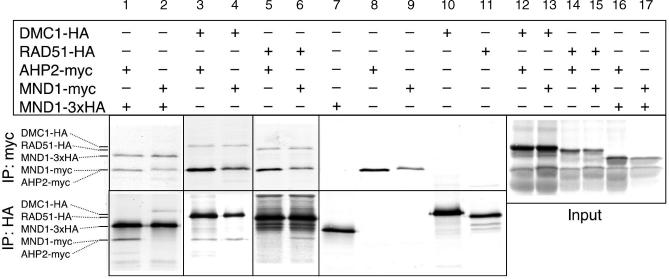
AtMND1 and AHP2 Interact with AtDMC1 and AtRAD51 In Vitro Coupled transcription/translation of AtMND1, AHP2, AtRAD51, and AtDMC1 was performed either alone or in the indicated combinations, in the presence of radioactively labeled L-methionine. Samples were split and incubated with one of the antibodies, directed against the c-myc or the HA epitope tag. The protein bands corresponding to AtMND1, AHP2, AtRAD51 and AtDMC1 are indicated. The first six lanes correspond to coimmunoprecipitation experiments, the following five lanes correspond to controls, demonstrating the specificity of the immunoprecipitations, and the last six lanes correspond to the cotranslated protein samples before immunoprecipitation.

We addressed the issue of direct interaction between the RecA-related proteins—AtRAD51 and AtDMC1—and AtMND1/AHP2 by carrying out in vitro protein interaction studies with proteins produced by in vitro transcription and translation. We demonstrated binding of AtMND1 and AHP2 (Figure 9, lane1), as anticipated in our previous study, in which this interaction was observed in a yeast two-hybrid system study [28]. AtMND1 also interacted with itself, but the coimmunoprecipitation of an AtMND1–AtMND1 complex was only possible with one of the two epitope tags used (Figure 9, lane 2). Interactions of AtMND1 with itself have also been reported in a yeast two-hybrid assay (unpublished data). Both AtMND1 and AHP2 interacted with AtDMC1 (Figure 9, lanes 3 and 4). In addition, both AHP2 and AtMND1 interacted with AtRAD51 (Figure 9, lanes 5 and 6). While the AHP2-AtRAD51 interaction was detected with only one of the two epitope tags used (Figure 9, lane 5), the interaction of AtMND1 with AtRAD51 was detected with both of the two epitope tags (Figure 9, lane 6).

## Discussion

The interaction of Mnd1 with Hop2 during meiotic recombination is conserved in yeasts, plants, and mammals. Several lines of evidence have shown that the Mnd1–Hop2 complex cooperates with Dmc1 [1,22] in yeast and mammals. Only in vitro assays have indicated that the mammal Mnd1–Hop2 complex may also interact functionally with Rad51 [19–21,47]. Little is currently known about the role in plants of the interplay between the Mnd1–Hop2 complex and RecA-related recombinases during DNA strand exchange processes, which are essential for meiotic recombination. We analyzed the genetic and physical interactions between Atmnd1, Atdmc1, Atrad51, and Atxrcc3 in the model plant *A thaliana* during meiotic recombination, focusing particularly on AtMND1.

### Conservation of the Mnd1 Function

Immunocytological analysis showed the AtMND1 protein to be extensively distributed on chromatin, throughout prophase I. The loading of AtMND1 onto chromosomes is independent of meiotic recombination, axial element formation, and cohesion. However, the presence of AHP2, the Hop2 homolog [29], is essential for the correct distribution of AtMND1. The results of studies in S. cerevisiae are consistent with these observations, as a regular distribution of Mnd1 in this organism requires Hop2, but does not depend on DSB formation, axial element formation, and synaptonemal complex [12,17]. Mnd1 and Hop2 interact in yeasts and mammals [12,16,19,20,47,48]. Interaction between AtMND1 and AHP2 in yeast two-hybrid assays [28] and in vitro assays (this study), and the AHP2-dependence of the distribution of AtMND1, provide strong evidence for a conserved role of the Mnd1-Hop2 complex.

It remains unclear whether Mnd1 in yeast is localized on chromatin in the form of numerous foci [17] or covers the chromatin entirely [12,48]. Our study presents the first images of Mnd1 distribution in a higher eukaryote. We observed AtMND1 labeling along the entire length of chromosomes, with interspersed regions of higher staining intensity. Studies in yeast showed that Mnd1 is not present at sites of recombination, as this protein was not colocalized with Rad51 or Dmc1 [17]. Our observations indicate that there is no significant colocalization of AtMND1 with AtDMC1, as AtDMC1 foci are seen only in regions labeled with diffuse AtMND1 staining and not in regions with more pronounced focus-like staining. This result confirms that AtMND1 may not be concentrated at sites of recombination, demonstrating another aspect of the conservation of Mnd1 function from yeast to plants.

### Synapsis Is Similarly Impaired in *Atmnd1*, *Atrad51*, *Atdmc1*, and *Atxrcc3*

In a previous study, we described the meiotic phenotype of the *Atmnd1* mutant [28], and showed that the typical zygotene and pachytene stages are absent in *Atmnd1*, and are replaced by a failed pachytene stage characterized by the absence of extended synapsis. We show here that this asynaptic phenotype is consistent with an aberrant distribution of AtZYP1, the central element of the SC [45]. In *Atmnd1* mutants, AtZYP1 is localized to only a few foci, some of which extend over short stretches. In contrast, synapsis is almost complete in the *mnd1* mutant of S. cerevisiae [17,49] and this synapsis is presumably nonhomologous, as shown for the *hop2* mutant [14]. Limited, mostly nonhomologous synapsis is observed in mouse *Hop2* mutant spermatocytes [15]. We infer that there is a conserved involvement of the Mnd1–Hop2 complex in SC formation.

The asynaptic phenotype of *Atmnd1* mutants has also been observed in other *Arabidopsis* mutants, such as the *Atrad51* and *Atrad51C* mutants [25,32], and we show that the *Atdmc1* and *Atxrcc3* mutants also lack typical pachytene stages. Consistent with these observations, we show that the distribution of AtZYP1 in the *Atrad51*, *Atdmc1*, and *Atxrcc3* mutants is similar to that observed in *Atmnd1* mutants [45] (this study). All four genes seem to be necessary for SC assembly. Limited synapsis may occur even in the absence of the two most prominent recombinases, AtRAD51 and AtDMC1. In contrast, ZYP1 foci do not form in the *Atspo11–1* mutant [45]. This shows that the limited extent of AtZYP1 staining in all the mutants analyzed may not be dependent on the strand invasion mediated by the recombinases, while it depends on DSB formation.

### *AtMND1*, *AtRAD51*, and *AtXRCC3* Act during the Same Step of Meiosis

Cytological analysis of *Atmnd1*, *Atrad51*, and *Atxrcc3* showed that all three mutants displayed the same meiosis I–related defects, characterized by entangled masses of chromatin at metaphase I and DNA fragmentation at anaphase I. In all three mutants, fragmentation is abolished by the depletion of AtSPO11–1 [25,28,50], a protein catalyzing meiotic DSB formation [23]. The observation of identical phenotypes in the associated double mutants is consistent with these proteins acting during the same step in meiosis. All three mutants display connections between multiple chromosomes at metaphase I, suggesting prior interactions between nonhomologous chromosomes. It has been suggested that such connections may result from the DSB repair mediated by the nonhomologous end-joining (NHEJ) machinery [27]. However, the mutation of *AtKU80*, a gene involved early in NHEJ [51], changes neither the *Atmnd1* nor the *Atrad51* phenotype (Julien Vignard, Raphael Mercier, unpublished results), suggesting that these connections between multiple chromosomes are not generated by NHEJ.

### AtRAD51 and AtXRCC3 Cooperate for Sister Chromatid–Mediated DSB Repair in the *Atdmc1* Mutant

The chromosomes of *Atdmc1* mutants neither associate in metaphase I, nor fragment in anaphase I. Instead, ten univalents are visible at metaphase I. It appears that DSBs occur normally in this mutant but are repaired using the sister chromatid as a template [26,27], as revealed by the fragmentation seen in *Atdmc1* mutants when *AtRAD51* is silenced [27] or in the *Atrad51-Atdmc1* double mutant (this study). One function of AtDMC1 may therefore be to prevent DSB repair between sister chromatids, or to favor interhomolog repair. In contrast, AtRAD51 may initiate homology searches regardless of the target (sister chromatid or the chromatids of the homologous chromosome). This is consistent with the known role of Rad51 in yeast and mammals during mitotic recombination [52] and the preference of Rad51 for inter-sister homologous recombination in somatic cells [53,54] or in yeast meiotic cells when Dmc1 is depleted [55]. The function of Dmc1 in driving the meiotic recombination towards the homolog seems to be conserved in yeast and plants. However, efficient Rad51-dependent inter-sister recombination in the absence of Dmc1 occurs in *Arabidopsis*, but not in yeast [55]. Nevertheless, the depletion of Red1, a major structural component of meiotic chromosomes [56], in *S. cerevisiae dmc1* mutant strains, facilitates the repair of meiotic DSB by Rad51-mediated recombination between sister chromatids [55]. *Arabidopsis* seems to have no equivalent function for preventing AtRAD51-dependent inter-sister chromatid DSB repair in *Atdmc1*. No Red1 homolog has been found in plants. Thus, inter-sister DSB repair mediated by AtRAD51 in the absence of AtDMC1 in *Arabidopsis* may not be restricted by a meiotic chromosome structural component as in budding yeast. Similarly, in yeast, the activity of Rad51 is downregulated by Hed1 when Dmc1 is unavailable [57]. It seems that such a regulator does exist in *Arabidopsis*, and no Hed1 homolog has so far been identified in plants.

The *Atrad51* and *Atxrcc3* mutations result in similar meiotic defects, markedly different from those associated with the *Atdmc1* mutation. In this study, we have shown that the meiotic phenotype of *Atdmc1-Atxrcc3* and *Atdmc1-Atdrad51* double mutants is equivalent to the phenotype of *Atxrcc3* and *Atrad51* single mutants, demonstrating that both AtRAD51 and AtXRCC3 are required for the sister chromatid mediated DSB repair in the *Atdmc1* mutant. These results suggest that *AtRAD51* and *AtXRCC3* act at the same step of meiotic recombination during strand invasion and that their functions are not redundant. Consistent with this hypothesis, the human Xrcc3 protein interacts physically with Rad51 [58]. Many studies have shown that Xrcc3 form a complex with Rad51C, and that this complex may contain Rad51 [10,59,60]. Two-hybrid analyses performed with *Arabidopsis* sequences confirmed that AtXRCC3 interacts with AtRAD51 [34]. These results indicate that the AtRAD51 and AtXRCC3 proteins cooperate in the same recombination step during meiosis

### AtMND1 Functionally Interacts with AtRAD51

Interestingly, the *Atmnd1-Atdmc1* double mutant displayed meiotic defects intermediate between those of the single mutants. This intermediate phenotype is dependent on AtRAD51 and AtXRCC3. We assume that the efficient repair of DSB via the sister chromatid in the *Atdmc1* mutant requires not only AtRAD51 and AtXRR3, but also AtMND1. It should be noted that although meiosis I defects of *Atmnd1* are largely abolished in the *Atdmc1* mutant background, meiosis II defects are so severe that the overall low fertility of the double mutant resembles that of *Atmnd1* single mutants. We infer that AtRAD51 is required to mediate inter-sister chromatid DSB repair (see above), and that this process is promoted by the presence of AtMND1 in an *Atdmc1* mutant background.

We therefore investigated the possibility of a direct interaction between *AtMND1* and *AtRAD51* and showed this interaction in vitro. Biochemical studies in mammals have demonstrated that the Mnd1–Hop2 complex stimulates the strand exchange activity of Dmc1 or Rad51 [19,20]. Whether Mnd1–Hop2 affect Rad51 activity in yeast has not been reported. Organisms lacking *MND1* and *HOP2* orthologs, such as nematodes, fruit flies, and Neurospora crassa, also have no *DMC1*, indicating that the role of Mnd1-Hop2 may be restricted to a Dmc1-dependent pathway [61]. We provide here the first demonstration of genetic interactions between Mnd1 and Rad51. We also provide evidence that both AtMND1 and AHP2 interact with AtRAD51 and AtDMC1 in vitro. However, we cannot exclude the possibility that in vivo, in the presence of AtDMC1, *AtMND1* may not interact with *AtRAD51* due to the higher affinity of the AtMND1–AHP2 complex for AtDMC1.

### AtDMC1 Foci Depend on *AtRAD51* and *AtXRCC3* and Accumulate in *Atmnd1*

In *S. cerevisiae mnd1* and *hop2* mutants, Rad51 and Dmc1 foci accumulate for longer and in greater numbers than in the wild type [12,14,17]. In *hop2* knockout mice, both Rad51 and Dmc1 foci are formed and accumulate along the chromosomal cores [15]. It therefore appears that DSBs are not repaired and that recombinase focus disassembly is impaired in *mnd1* and *hop2* mutants. We show here that AtDMC1 foci are formed in *Atmnd1* mutants and accumulate in greater numbers than in the wild type. We cannot exclude the possibility that more DSB are formed in the mutant than in the wild type. However, it seems more likely that, as in yeast and mammals, the absence of AtMND1 in *Arabidopsis* leads to the failed repair of meiotic DSB, characterized by a defect in AtDMC1 focus disassembly.

In contrast, *Atrad51* and *Atxrcc3* mutants have far fewer AtDMC1 foci than the wild type. Thus, the normal distribution of AtDMC1 requires AtRAD51 and AtXRCC3. It has been shown in S. cerevisiae that the normal distribution of Dmc1 requires *RAD51* [62,63]. Less is known about the possible requirement of Xrcc3 for the correct distribution of Dmc1. However, Rad51 foci do not form in the absence of Xrcc3 in somatic cells from Chinese hamster, chicken, and human [64–66], suggesting that Xrcc3 may indirectly interfere with the distribution of Dmc1 at meiosis. AtDMC1 foci may be formed in smaller numbers in the mutants, or their turnover may be faster than in the wild type, possibly due to an unstable AtDMC1 filament structure in the absence of AtRAD51 and AtXRCC3.

### AtXRCC3 Is Dispensable for AtDMC1 Loading in an *Atmnd1* Mutant Background, Whereas AtRAD51 Is Not

This study is the first to dissect the functional differences between AtRAD51 and AtXRCC3 during meiosis, demonstrating that AtXRCC3 is dispensable for AtDMC1 foci formation in an *Atmnd1* mutant background, whereas AtRAD51 is not. It has been suggested that the Rad51C–Xrcc3 complex in mammals may be associated with the Holliday junction resolvase complex and may play a role in the resolution of recombination intermediates [25,67]. We report here another aspect of the different roles of AtRAD51 and AtXRCC3 in meiosis*,* which became apparent in the *Atmnd1* mutant background. According to one model consistent with our data, AtRAD51 plays a crucial role in loading AtDMC1 onto the processed DSB ends to form a nucleoprotein filament. AtMND1 and its partner AHP2 are subsequently required for efficient homology searches. If AtMND1 is depleted, the turnover rate of this process is much lower, resulting in a higher AtDMC1 focus count. AtXRCC3 may be required to stabilize AtDMC1-containing nucleoprotein filaments. When AtXRCC3 is absent, the nucleoprotein filaments are rapidly disassembled, resulting in a lower AtDMC1 focus count. Consistent with this model, the number of AtDMC1 foci in *Atmnd1-Atxrcc3* double mutants was found to be smaller than that in *Atmnd1* single mutants, but greater than that in *Atxrcc3* single mutants. However, this model conflicts with previous findings that Xrcc3 is required for the correct distribution of RAD51 in mammalian somatic cell culture [64–66], and with observations that Xrcc3 is recruited to DSB sites earlier and independently of Rad51 in human somatic cells [68]. Further studies, notably analyzing the distribution and number of AtRAD51 foci in various mutant backgrounds during meiosis, will be required to decipher this apparent divergence between mammalian somatic recombination and meiosis in *Arabidopsis*.

## Materials and Methods

### Plant material.

The wild-type reference plant material used in this study was A. thaliana accession Columbia (Col-0). The mutants used were *Atmnd1* [28], *Atrad51* [25], *Atdmc1* [26], *Atxrcc3* [31], *ahp2* [29], *Atspo11-1-2* [23], *Atrec8*/*dif* [40], *Atscc3–1* [41] *swi1–2* [43], and *Atmre11* [39].

### Growth conditions.

*Arabidopsis* plants were cultivated in a greenhouse or growth chamber under the following conditions: photoperiod 16 h day/8 h night; temperature 20 °C day and night; humidity 70%.

### Genetic analyses.

Double mutants were obtained by crossing plants heterozygous for each mutation. Double heterozygous plants identified in the F1 generation were self-fertilized to obtain the F2 generation. Plants in the F2 generation homozygous for the two relevant mutations were analyzed. The *Atrad51-Atdmc1-Atmnd1* and *Atdmc1-Atmnd1-Atxrcc3* triple mutants were obtained by crossing *Atrad51-Atdmc1* and *Atmnd1-Atxrcc3* double heterozygous plants from the F2 generations. Quadruple heterozygous plants were self fertilized and triple homozygotes in their offspring were identified and analyzed. Plants were selected by the PCR genotyping of individual plants, using diagnostic primer sets.

### Microscopy.

Comparisons of the development of pollen mother cells were carried out as described by Grelon et al. [23]. Prophase-stage spreads for immunocytology were prepared as described by Armstrong et al. [35], incorporating the modifications described by Chelysheva et al. [41].

All observations were made with a LEICA DM RXA2 microscope using an oil PL APO 100X/1.40 objective (Leica); photographs were taken using a CoolSNAP HQ (Roper) camera driven by Open LAB 4.0.4 software; all images were further processed with Open LAB 4.0.4 or AdobePhotoshop 7.0.

### Antibodies.

The polyclonal anti-ASY1 antibody has been described elsewhere [35], and was used at a dilution of 1:500. The polyclonal anti-AtZYP1 antibody has been described elsewhere [45]. It was used at a dilution of 1:500. The purified polyclonal anti-AtDMC1 antibody was described by Chelysheva et al. [46]. It was used at a dilution of 1:20.

The full-length coding region of AtMND1 was inserted into the expression vector pET29 (Novagen) as an N-terminal fusion to a HIS tag. The resulting construct was transferred into E. coli BL21 cells (Novagen). Purified recombinant protein was prepared as previously described [41] and used to produce a rat polyclonal antiserum (Eurogentec). This antibody was used at a dilution of 1:200. The specificity of the antibody was tested by preincubation serum, a dilution of 1:100 with 5 μg/mL of purified recombinant AtMND1 protein, or 5 μg/mL of BSA for 1h at 4 °C prior to its use for cytology and western blotting experiments.

Secondary antibodies used were goat anti-rabbit AlexaFluor488, goat anti-rabbit AlexaFluor566, goat anti-rat AlexaFluor488, and goat anti-rat AlexaFluor566 (Invitrogen). Secondary antibodies were used at a 1:100 dilution.

### Vector construction.

We constructed an AtMND1-HA fusion, by amplifying the *AtMND1* cDNA in plasmid U50561 by PCR, using Pfu-polymerase (MBI Fermentas) and the primers SpeI_up (5′-TAACTAGTTCTAAGCTTCATCTTGTACTAGC-3′) and SmaI_dn (5′-ATCCCGGGATGTCTAAGAAACGGGGACTTTC-3′). This fragment was then ligated into pCR2.1.TOPO (Invitrogen). The *AtMND1* cDNA was released by digestion with SpeI and SmaI and ligated into pCR2.1/3xHA digested with the same enzymes, giving rise to pCR2.1/MND1-3xHA (or MND1-3xHA for short), which encodes the HA tag fused to the N terminus of AtMND1 under the control of a T7 promoter.

A vector encoding AtMND1 fused to a GAL4 DNA-binding domain was constructed by amplifying the *AtMND1* cDNA contained within plasmid U50561 by PCR, using Ex-Taq (TaKaRa) and the primers M1 (5′-CATATGTCTAAGAAACGGGGACTTTC-3′) and M4 (5′-CTAAGCTTCATCTTGTACTAGC-3′). The resulting fragment was ligated into pCR2.1.TOPO (Invitrogen). The cDNA was obtained from this vector by digestion with *Nde*I/*Bam*HI, and was ligated into pGBKT7 (Clontech) digested with the same enzymes, giving rise to the Y2H vector pGBKT7/AtMND1 (or MND1-myc).

For construction of the AHP2-GAL4 DNA binding domain fusion, the *AHP2* cDNA in pGAD10/AHP2 [29] was amplified by PCR (Ex-Taq/TaKaRa) with the primers AHP2_Ncodn (5′-TAGTCCATGGCTCCTAAATCGGATAAC-3′) and AHP2_PstIup (5′-TATGCTGCAGTTACTGTCCTCGAGGCCTC-3′). The PCR fragment was digested with the restriction enzymes NcoI and PstI and ligated into pGBKT7 digested with the same enzymes, giving rise to the Y2H vector pGBKT7/AHP2 (or AHP2-myc). All fragments generated by PCR were sequenced to confirm error-free amplification.

### In vitro transcription/translation and coimmunoprecipitation.

Antibodies were coupled to Protein G beads, by washing 30 μl of Protein G Sepharose (Amersham) three times with NET2 buffer (150 mM NaCl, 50 mM Tris-Cl pH7.4, 0.05% Igepal), incubating overnight at 4 °C with 300 μl of undiluted sera containing antibodies against the c-myc (#9E11) or the HA tag (12CA5) (both antibodies were generously provided by K. Nasmyth), and then washing three times with NET2 buffer. Vector DNA (1 μg) was used as a template for in vitro translation with the TNT Coupled Reticulocyte Lysate System (Promega), as recommended by the manufacturer. One third of the reaction mixture was incubated for 2 h at 4 °C with myc-coupled beads, one third was incubated with HA-coupled beads, and one third was kept untreated. The beads were washed three times with buffer, resuspended in 10 μl 2× Laemmli sample buffer, and boiled at 95 °C for 5 min. The sample was briefly centrifuged in a microfuge and the supernatant was run on a 12% polyacrylamide gel. The gel was incubated for 30 min in 20% methanol/10% acetic acid with gentle shaking, followed by 15 min in acetic acid and 15 min in 24% diphenoloxazol (in acetic acid). The gel was then floated on dH_2_O, washed three times with dH_2_O for 5 min each, then incubated for 5 min with 3% glycerol and vacuum dried for at least 3 h at 60 °C.

## Supporting Information

Figure S1 Localization of AtMND1 in a Series of Meiotic Mutants Male meiocytes from the *Atmre11* (A), *Atrad51* (B), *Atdmc1* (C), *Atrad51-Atdmc1* (D), *Atxrcc3* (E), *Atscc3* (F), *swi1* (G), and *asy1* (E) mutants. Chromosomes are stained with the ASY1 antibody (red, A–G) or the AtSCC3 antibody (H) and the AtMND1 antibody (A–H). As in the wild type, the AtMND1 signal is visible along the length of the chromosome at prophase I stages in all mutants. Scale bar, 10 μm.
(71.5 MB TIF)

Figure S2 Absence of Synapsis at Pachytene in *Atrad51*, *Atmnd1*, *Atdmc1*, *Atxrcc3*, and *Atrad51-Atdmc1* Mutants During wild-type pachytene stages (A), chromosomes are fully synapsed. In *Atrad51* (B), *Atmnd1* (C), *Atdmc1* (D), *Atxrcc3* (E), and *Atrad51-Atdmc1* (F) mutants, chromosomes do not synapse. Scale bar, 10 μm.
(12.6 MB TIF)

Figure S3 Metaphase I–Anaphase I Defects in *Atrad51* and *Atxrcc3* Mutants In *Atrad51* (A, B) and *Atxrcc3* (C, D) mutants, an entangled mass of chromosomes is observed at metaphase I, with chromatid connections between multiple chromosomes (A, C). Segregation at anaphase I leads to chromosome fragmentation (B, D). Scale bar, 10 μm.
(7.9 MB TIF)

### Accession Numbers

The National Center for Biotechnology Information (NCBI) GenBank (http://www.ncbi.nlm.nih.gov/GenBank) accession number for plasmid U50561 is BT005435.

## Abbreviations

DSB: double-strand break
NHEJ: nonhomologous end-joining
SC: synaptonemal complex
